# Illness acceptance, health perception, and hopelessness levels in stroke survivors: A cross-sectional and correlational study

**DOI:** 10.15649/cuidarte.4588

**Published:** 2025-05-01

**Authors:** Sena Nur Yapar, Fatma Özkan Tuncay

**Affiliations:** 1 PhD student, Sivas Cumhuriyet University, Institute of Health Sciences, Internal Medicine Nursing, Sivas, Turkey. E-mail: senanuryapar@outlook.com Sivas Cumhuriyet University Sivas Turkey senanuryapar@outlook.com; 2 Assoc. Prof. Sivas Cumhuriyet University, Faculty of Health Sciences, Internal Medicine Nursing, Sivas, Turkey. E-mail: fozkan77@gmail.com Sivas Cumhuriyet University Sivas Turkey fozkan77@gmail.com

**Keywords:** Nursing, Stroke, Perception, Hope, Enfermería, Accidente Cerebrovascular, Percepción, Esperanza, Enfermagem, Acidente Vascular Cerebral, Percepção, Esperança

## Abstract

**Introduction:**

According to the World Health Organization, stroke is a clinical condition characterized by the sudden development of focal or global signs and symptoms in cerebral functions and is the second leading cause of death and the third leading cause of disability worldwide.

**Objective:**

This study aimed to determine the levels of illness acceptance, health perception, and hopelessness of stroke survivors and to examine the relationship between these variables.

**Materials and Methods:**

This study was conducted with 170 stroke survivors. Data were collected with the "Descriptive Information Form," "Modified Barthel Index," "Acceptance of Illness Scale," "Perception of Health Scale," and "Beck Hopelessness Scale." The data were analyzed using SPSS 21.0 software.

**Results:**

Participants showed moderate levels of illness acceptance and mild levels of hopelessness. The mean health perception score was 50.30±0.59. A significant relationship was found between age, education, employment status, living arrangements, illness duration, post-stroke deficits, independence level, illness acceptance, health perception, and hopelessness levels. The health perception and hopelessness levels of the participants decreased as their level of illness acceptance increased. Increasing health perception levels were also found to increase hopelessness levels.

**Discussion:**

When the literature is reviewed, there are studies that support our findings in stroke and other chronic illnesses, but there are also studies with different results.

**Conclusion:**

The study results draw attention to the levels of illness acceptance, health perception, and hope, which have not been discussed much but have important effects on the illness and rehabilitation process.

## Resumen

**Introducción:**

Según la Organización Mundial de la Salud el ictus es una afección clínica caracterizada por el desarrollo súbito de signos y síntomas focales o globales en las funciones cerebrales y es la segunda causa de muerte y la tercera de discapacidad en todo el mundo.

**Objetivo:**

Este estudio busca determinar los niveles de aceptación de la enfermedad, la percepción de salud y la desesperanza de los supervivientes de accidentes cerebrovasculares e investigar la relación entre estas variables.

**Materiales y métodos:**

Este estudio se realizó con 170 supervivientes de accidentes cerebrovasculares. Se recogieron datos con el «Formulario descriptivo», el «Índice Barthel modificado», la «Escala de aceptación de enfermedades», la «Escala de percepción de salud» y la «Escala de desesperanza de Beck». Los datos se analizaron con el programa SPSS 21.0.

**Resultados:**

Los participantes mostraron niveles moderados de aceptación de la enfermedad y niveles leves de desesperanza. La puntuación media de la percepción de salud fue de 50,30±0,59. Se encontró una relación significativa entre edad, educación, situación laboral, composición del hogar, duración de la enfermedad, déficits posictus y nivel de independencia, y entre la aceptación de la enfermedad, la percepción de salud y la desesperanza. Se observó que la percepción de salud y los niveles de desesperanza de los participantes disminuían a medida que aumentaba su nivel de aceptación de la enfermedad. También se observó que el aumento de los niveles de percepción de salud incrementaba los niveles de desesperanza.

**Discusión:**

Cuando se revisa la literatura, hay estudios que apoyan nuestros hallazgos en ictus y otras enfermedades crónicas, pero también hay estudios con resultados diferentes.

**Conclusiones:**

Con los resultados del estudio, se llama la atención sobre los niveles de aceptación de la enfermedad, la percepción de salud y la esperanza, de los cuales no se ha hablado mucho, pero que tienen efectos importantes en el proceso de enfermedad y rehabilitación.

## Resumo

**Introdução:**

De acordo com a Organização Mundial da Saúde, o AVC é uma condição clínica caracterizada pelo desenvolvimento súbito de sinais e sintomas focais ou globais nas funções cerebrais e é a segunda principal causa de morte e a terceira principal causa de incapacidade em todo o mundo.

**Objetivo:**

Neste estudo, o objetivo foi determinar os níveis de aceitação da doença, percepção da saúde e desesperança dos sobreviventes de AVC e investigar a relação entre as variáveis.

**Materiais e métodos:**

Este estudo foi realizado com 170 sobreviventes de AVC. Os dados foram coletados com o “Formulário de Informações Descritivas”, o “Índice de Barthel Modificado”, a “Escala de Aceitação da Doença”, a “Escala de Percepção da Saúde” e a “Escala Beck de Desesperança”. Os dados foram analisados com o software SPSS 21.0.

**Resultados:**

Os participantes apresentaram níveis moderados de aceitação da doença e níveis leves de desesperança. A pontuação média de percepção da saúde foi de 50,30±0,59. Foi encontrada uma relação significativa entre idade, escolaridade e situação de trabalho, coabitantes, duração da doença, déficits pós-AVC e nível de independência e níveis de aceitação da doença, percepção da saúde e desesperança. Verificou-se que os níveis de percepção de saúde e desesperança dos participantes diminuíram à medida que o nível de aceitação da doença aumentou. O aumento dos níveis de percepção da saúde também aumentou os níveis de desesperança.

**Discussão:**

Quando a literatura é revisada, há estudos que apoiam nossos achados no AVC e em outras doenças crônicas, mas também há estudos com resultados diferentes.

**Conclusões:**

Com os resultados do estudo, chama-se a atenção para os níveis de aceitação da doença, percepção da saúde e esperança, que não foram muito discutidos, mas que têm efeitos importantes sobre a doença e o processo de reabilitação.

## Introduction

According to the World Health Organization (WHO), stroke is a clinical condition characterized by the sudden development of focal or global signs and symptoms in cerebral functions, persisting for at least 24 hours or longer or resulting in death due to impaired cerebral perfusion with no apparent cause other than vascular origins^1^. Although stroke incidence varies across countries, it is recognized as a major global public health concern^2^. According to WHO, stroke is the second leading cause of death and the third leading cause of disability worldwide, following ischemic heart disease^3^. The incidence and impact of stroke are estimated to increase in developing countries due to population aging and demographic shifts^4^.

After a stroke, individuals continue their lives with severe disabilities, including neuromotor and sensory deficits, decreased muscle tone, and impairments in speech, hearing, vision, and balance^2^,^5^. These disabilities affect individuals' independence and activities of daily living (ADLs), making it difficult to cope with, adapt to, and accept the illness^5^,^6^. Stroke management requires individualized nursing care, given individuals' different symptoms and clinical findings. The main goals of nursing care for stroke patients are to support coping with the illness, increase independence, and improve quality of life^2^,^5^.

Illness acceptance, which is the first step in coping with the illness, significantly affects the quality of life, recognition of limitations, self-worth, self-control, and general health status^7^. Studies in the literature show that illness acceptance among individuals with chronic illnesses has positive effects on effective coping strategies, depression reduction or prevention, positive health perception, quality of life, self-care activities, and illness management^8^–^11^. A review of the literature reveals studies reporting that illness acceptance contributes to psychological resilience and relaxation, development of positive health behaviors, adherence to treatment, and positive thoughts about the future^12^–^14^. Illness acceptance is important for the holistic management of chronic illness. Therefore, nursing interventions that support and improve illness acceptance should be assessed and integrated into psychosocial care strategies^15^.

Illness acceptance influences health behaviors by increasing motivation to adopt desired behaviors. Assessing health behaviors is important in conditions like stroke, which impact many dimensions of life^16^. While health behaviors affect health outcomes, beliefs, attitudes, and health perceptions also play a role in developing these behaviors^17^. Health perception is a subjective indicator that allows individuals to assess their feelings, thoughts, and expectations regarding their health in biological, psychological, and social terms. It has an important place in developing, maintaining, and improving health behaviors among individuals with chronic illnesses. Chronic illnesses cause psychological distress in individuals, and the individual's health perception serves as a mediating step between chronic illness and psychological distress. In this process, nurses should consider how individuals define their health, which phenomena they relate to health, and their health perception^18^,^19^. Another main variable influencing health behaviors and treatment adherence is the individual's level of hope^20^.

Hopelessness, a recognized nursing diagnosis, affects an individual’s physical, psychological, and social health^21^. Hopelessness is the attitude of individuals who believe that positive situations will not occur in the future and that unfavorable situations will continue to develop^20^. Worsening health conditions and post-stroke functional disabilities can lead to hopelessness^22^. Nurses play a crucial role among healthcare professionals in providing support to individuals experiencing hopelessness, helping them develop coping mechanisms by activating social support factors^23^. Anastasiades et al. reported that depression and hopelessness are the most important factors affecting adherence to drug treatment and health behaviors^24^.

Illness acceptance, health perception, and future expectations may be among the factors affecting health behaviors, illness adaptation, and adherence to stroke treatment. However, there is a limited number of studies in the literature examining the relationship between these variables in stroke survivors, and no relevant study has been conducted in Turkey^22^,^25^. In addition to clinical assessment, assessing illness acceptance, health perception, and hopelessness in stroke survivors would help them maintain self-care management and control the illness more effectively. Therefore, the results of this study would contribute to the literature. Additionally, this study could serve as a guide for determining factors influencing illness acceptance, health perception, and hopelessness levels in stroke survivors and planning targeted nursing care.

## Materials and Methods

**Study design**

This cross-sectional and correlational study was conducted between September 2021 and February 2022 to study the levels of illness acceptance, health perception, and hopelessness in stroke survivors and to explore the relationship between these variables.

**Study hypotheses**

H1: There is a difference in illness acceptance, health perception, and hopelessness levels of stroke survivors according to sociodemographic variables (one-way hypothesis).

H1: There is a relationship between illness acceptance, health perception, and hopelessness levels in stroke survivors (one-way hypothesis).

**Sample**

The population comprised individuals diagnosed with stroke who sought care at the neurology polyclinic of a state hospital in Sivas, Turkey. The sampling rate was 10%, with a margin of error of 0.05, and the minimum required sample size was calculated as 167 individuals (0.95 confidence)^26^. In case of incomplete or incorrectly completed forms, 170 participants were recruited, and all individuals completed the study. The study included individuals who had experienced a stroke at least three months prior, had no communication problems that would hinder their ability to respond to the questions, and agreed to participate in the study after being informed. Individuals with a history of reinfarction and comorbidities affecting consciousness were excluded.

**Data collection tools**

*Descriptive Information Form:* This form, developed by the researcher based on the literature, consists of 13 questions—seven addressing sociodemographic characteristics and six exploring health behaviors and illness-related characteristics^22^,^25^.

*Modified Barthel Index (MBI):* It was developed by Barthel and Mahoney and is used for assessing ADLs^27^. The Turkish adaptation was validated by Küçükdeveci et al. with a Cronbach's alpha of 0.83^28^. The score ranges from 0 to 100 (0-20: totally dependent, 21-61: severely dependent, 62- 90: moderately dependent, 91-99: slightly dependent, and 100: Completely independent). In this study, Cronbach's alpha was 0.73.

*Acceptance of Illness Scale (AIS):* It was developed by Felton et al. and adapted into Turkish by Besen and Esen^29^,^30^, with a Cronbach's alpha of 0.79. The scale consists of items rated on a 5-point Likert scale (1= strongly agree, 5= strongly disagree), with item 6 reverse-scored. The total score ranges from 8 to 40 (8-19: low, 20-30: moderate, and 31-40: high illness acceptance). In this study, Cronbach's alpha was 0.90.

*Perception of Health Scale (PHS):* It was developed by Diamond et al. and adapted into Turkish by Kadıoğlu and Yıldız^31^,^32^, with a Cronbach's alpha of 0.77. The scale consists of four sub-dimensions: center of control, self-awareness, certainty, and importance of health. Positive statements (items 1, 5, 9, 10, 11, and 14) are scored on a 5-point Likert scale: "5 = strongly agree, 1 = strongly disagree."Negative statements (items 2, 3, 4, 6, 7, 8, 12, 13, and 15) are reverse-scored. The total score ranges from 15-75 with no defined cut-off point. Higher scores indicate a higher health perception. In this study, Cronbach's alpha was 0.71 with sub-dimensions values of 0.68, 0.82, 0.75, and 0.72, respectively).

*Beck Hopelessness Scale (BHS):* It was developed by Beck et al. and adapted into Turkish by Durak and Palabıyıklıoğlu^33^,^34^, with a Cronbach's alpha of 0.93. The scale consists of three sub-dimensions: feelings about the future, motivation, and future expectations. The total score ranges from 0 to 20 (0-3: none, 4-8: mild, 9-14: moderate, and >14: severe hopelessness). In this study, Cronbach's alpha was 0.91, with sub-dimension values of 0.77, 0.83, and 0.64, respectively).

**Data collection procedure**

Before the study, the researchers obtained the required permissions from the institution where the research was conducted and ethical approval from the ethics committee. Written and verbal informed consent was obtained from all participants. Participants who could read the questions completed the forms alone, while the researchers assisted those who were illiterate or visually impaired. The researchers completed the MBI based on information provided by both participants and caregivers.

**Ethical considerations**

To conduct the study, Approval No: 2021-06/30 was obtained from the Cumhuriyet University Non-Interventional Clinical Research Ethics Committee, along with written authorization from the hospital administration where the study was conducted. Participants were informed that their data would remain confidential and be used only for research purposes. The research was conducted in accordance with the principles of the Helsinki Declaration.

**Data analysis**

Data were analyzed using SPSS 21.0 software. The Kolmogorov-Smirnov test was used to assess the normality of the scale scores. Since the data were not normally distributed, the Mann-Whitney U test was applied for comparing two independent variables, and one-way ANOVA and Kruskal- Wallis tests were applied for comparisons among multiple variables (p<0.05). When the ANOVA test revealed a significant difference, the LSD post hoc test was applied to determine which groups differed. Mann-Whitney U pairwise comparison test was conducted for the Kruskal-Wallis H test. Spearman's Rho correlation test was used to analyze correlations between the scales. A 95% confidence interval was applied (p<0.05). Linear regression analysis was conducted to assess relationships between variables (p<0.05). Two regression models were developed, each considering illness acceptance and hopelessness as independent variables. The study's dataset is available for free access and consultation on Mendeley Data^35^.

## Results

Table 1 shows the demographic characteristics of the participants. It was determined that 52.40% (n = 89) of the participants were male, and 34.10% (n = 58) were between 71 and 80 years old. Additionally, 54.70% (n = 93) had completed primary education, 67.10% (n = 114) were married, 93.50% (n = 159) were unemployed, and 66.50% (n = 113) lived with their families. Regarding stroke- related information, 43.9% (n = 79) of the participants had experienced their stroke 3-6 months prior, 58.90% (n = 106) had one or more post-stroke deficits, and 35.30% (n = 60) were classified as moderately dependent. 

**Table 1:** Demographic Characteristics of Participants

Characteristics	n (170)	%
Gender		
Female	81	47.60
Male	89	52.40
Age groups		
≤60	30	17.60
61-72	44	25.90
71-80	58	34.10
≥81	38	22.40
Education		
Illiterate	59	34.70
Elementary school	93	54.70
High school	18	10.60
Marital status		
Single	56	32.90
Married	114	67.10
Employment		
Employed	11	6.50
Unemployed	159	93.50
Living arrangements		
With family	113	66.50
With a son/daughter	41	24.10
Alone	16	9.40
Illness Duration		
3-6 months	79	43.9
6-12 months	34	18.9
>12 months	67	37.2
Post-stroke deficits		
No deficits	74	41.1
One deficit	76	42.2
More than one deficit	30	16.7
MBI	(81.96±21.78)	
Totally dependent	5	2.90
Severely dependent	23	13.50
Moderately dependent	60	35.30
Slightly dependent	42	24.70
Completely independent	40	23.50

MIB: Modified Barthel Index

Table 2 shows the total mean scores of the scales. The mean total score for the AIS was 24.78 ± 0.61, while the mean total score for the PHS was 50.30 ± 0.59, with sub-dimension scores as follows: importance of health (10.78 ± 0.21), center of control (17.89 ± 0.34), self-awareness (9.60 ± 0.19), and certainty (12.01 ± 0.27). The mean total score for the BHS was 7.80 ± 0.41, with sub-dimension scores as follows: feelings about the future (2.7 5± 0.15), motivation (2.40 ± 0.17), and future expectations (2.42 ± 0.10). 

**Table 2:** AIS, PHS, and BHS total mean scores

Scales	x ±SD	Min-Max
AIS	24.78 ± 0.61	8-40
PHS	50.30 ± 0.59	19-75
Importance of health	10.78 ± 0.21	3-15
Center of control	17.89 ± 0.34	6-25
Self-awareness	9.60 ± 0.19	3-15
Certainty	12.01 ± 0.27	4-20
BHS	7.80 ± 0.41	0-20
Feelings about the future	2.75 ± 0.15	0-7
Motivation	2.40 ± 0.17	0-7
Future expectations	2.42 ± 0.10	0-5

SD: Standard Deviation, AIS: Acceptance of Illness Scale, PHS: Perception of Health Scale, BHS: Beck Hopelessness Scale

Table 3 presents relationships between participants' scale total mean scores. A positive correlation was found between independence level and illness acceptance, while a negative correlation was observed between independence level and hopelessness (p<0.01). This indicates that as participants' independence increases, their illness acceptance rises, and their hopelessness decreases. Additionally, as participants' illness acceptance increases, their health perceptions and hopelessness levels decrease. In the study, a positive relationship was also identified between health perception and hopelessness (p<0.05), suggesting that as participants' health perception increases, their hopelessness levels also rise.

**Table 3:** Correlations between MBI, AIS, PHS, and BHS

Scales	2	3	4	5	6	7	8	9	10	11
1. MBI	.658**	-.049	-.051	-.239**	.417**	-.066	-.581**	-.502**	-.583**	-.456**
2. AIS	1	-.212**	-.152*	-.192*	.217**	-.257**	-.575**	-463**	-593**	-473**
3. PHS		1	,592**	.594**	.354**	.700**	.168*	.112	.190*	.140
4. Importance of health			1	.006	.415**	.201**	-.105	-.180*	-.028	-0.86
5. Center of control				1	-.299**	.230**	.429**	.419**	.369**	.374**
6. Self-awareness					1	.101	-.428**	-.477**	-.337**	-.382**
7. Certainty						1	.210**	.175*	.211**	.174*
8. BHS							1	.896**	.924**	.875**
9. Feelings about the future								1	.705**	.713**
10. Motivation									1	.733**
11. Future expectations										1

**p<0.01 *p<0.05 p-values based on Spearman’s Rho correlation test. MIB: Modified Barthel Index, AIS: Acceptance of Illness Scale, PHS: Perception of Health Scale, BHS: Beck Hopelessness Scale

Table 4 presents the relationships between demographic variables and scale mean scores. No significant differences were found in illness acceptance, health perception, and hopelessness levels based on gender (p>0.05). However, as age increased, levels of health perception (center of control and self-awareness sub-dimensions) decreased, while levels of hopelessness increased. A higher level of education was associated with greater illness acceptance and health perception scores (across all sub-dimensions) but with lower hopelessness scores (including all sub-dimensions). Married individuals had higher illness acceptance and health perception scores (particularly in the center of control, certainty, and self-awareness sub-dimensions) and lower hopelessness scores across all sub-dimensions. The study also found that employed individuals had higher illness acceptance and certainty sub-dimension scores and lower levels of hopelessness. Additionally, participants who lived with their families exhibited higher illness acceptance and health perception scores (specifically in the center of control and self-awareness sub-dimensions).

Table 4 also shows that individuals who had been living with the illness for 3-6 months exhibited higher levels of illness acceptance, as well as higher scores in the center of control and self-awareness sub-dimensions. In contrast, individuals with an illness duration of more than 6 months had higher levels of hopelessness across all sub-dimensions. A significant difference was found in illness acceptance, self-awareness sub-dimension, and hopelessness levels (across all sub-dimensions) according to the presence of post-stroke deficits (p<0.05). Individuals without post-stroke deficits had higher levels of illness acceptance and self-awareness scores, whereas those with post-stroke deficits exhibited higher levels of hopelessness across all sub-dimensions.

Table 5 presents the regression analysis of illness acceptance levels. MBI, PHS, and BHS levels affected AIS levels (p<0.05). MBI, PHS, and BHS levels explained 50% of the AIS variance. While individuals' independence levels positively impact illness acceptance, health perception and hopelessness levels negatively affect it. Additionally, Table 5 shows the regression analysis of BHS levels. Illness acceptance and independence levels had an effect on hopelessness (p<0.05). AIS and MBI levels explained 41% of the BHS variance. Independence and illness acceptance levels have a negative effect on the hopelessness level.

**Table 4:** Relationships between AIS, PHS, and BHS total mean scores and variables. x ± SD

	AIS	PHS					BHS			
		Importance of health	Center of control	Self- awareness	Certainty	Total	Feelings about the future	Motivation	Future expectations	Total
Gender										
Female	24.10±7.57	10.80±2.54	18.57±4.27	9.68±2.48	12.42±3.40	51.48±6.23	2.89±2.09	2.58±2.27	2.59±1.48	8.32±5.54
Male	25.42±8.48	10.77±2.96	17.26±4.67	9.52±2.67	11.63±3.65	49.19±8.76	2.62±2.00	2.23±2.21	2.26±1.34	7.30±5.13
p	0.272	0.772	0.073	0.705	0.128	0.062	0.439	0.206	0.142	0.262
Age groups										
≤60	28.70±9.28	47.87±7.92	10.83±2.72	13.97±5.08	10.07±2.83	12.97±3.67	5.10±4.60	1.43±1.72	1.53±1.83	2.10±1.49
61-70	29.05±9.83	46.16±8.23	11.50±2.35	12.43±4.36	10.45±2.24	11.70±3.64	6.91±5.41	2.52±1.92	2.25±2.44	2.14±1.55
71-80	24.57±8.55	42.81±7.62	10.50±2.92	11.71±4.13	9.24±2.71	11.34±4.11	7.76±4.74	2.79±1.91	2.57±2.35	2.41±1.26
≥81	19.95±8.29	41.82±7.49	10.39±2.95	10.68±4.62	9.00±2.74	11.89±4.07	11.21±5.37	4.00±1.90	3.97±2.64	3.26±1.55
p	**0.000**	**0.003**	0.266	**0.021 **	**0.049 **	0.128	** 0.000**	**0.000 **	**0.000 **	**0.002 **
Education										
Illiterate	22.97±8.23	41.00±7.56	10.12±2.94	10.34±4.29	8.83±2.79	11.76±3.91	10.00±5.19	3.56±1.84	3.44±2.53	2.97±1.40
Elementary	26.37±9.96	44.74±6.95	11.04±2.64	12.19±4.19	9.95±2.57	11.54±3.75	7.02±5.20	2.44±2.03	2.30±2.37	2.29±1.42
High school	28.61±10.16	53.28±8.20	11.72±2.42	17.06±3.56	10.78±2.07	13.72±4.51	5.00±4.74	1.72±1.84	1.56±2.15	1.83±1.82
p	**0.029 **	**0.000 **	**0.032 **	**0.000 **	**0.006 **	**0.025 **	**0.000 **	** 0.000**	**0.002 **	**0.002 **
Marital Status										
Single	21.29±8.66	41.32±7.87	10.45±2.92	11.04±4.82	8.80±2.70	11.02±4.16	10.25±5.01	3.68±1.77	3.52±2.53	3.05±1.43
Married	27.46±9.35	45.83±7.78	10.96±2.68	12.57±4.37	10.06±2.56	12.25±3.75	6.66±5.19	2.30±2.01	2.18±2.34	2.19±1.46
p	**0.000 **	**0.001 **	0.321	**0.020 **	**0.004 **	**0.043 **	**0.000 **	**0.000 **	**0.001 **	** 0.000**
Employment										
Employed	34.55±8	48.18±8.66	10.18±3.37	13.82±3.87	9.64±3.01	14.55±2.16	2.73±1.27	0.82±0.98	0.45±0.93	1.45±0.69
Unemployed	24.79±9.35	44.08±7.99	10.84±2.72	11.94±4.6	9.65±2.65	11.66±3.95	8.19±5.39	2.89±2.02	2.77±2.48	2.55±1.52
p	**0.001 **	0.103	0.708	0.132	0.845	**0.007 **	**0.000 **	**0.001 **	**0.001 **	**0.014 **
Living arrangements										
With family	27.29±9.46	45.69±7.83	10.98±2.68	12.48±4.41	10.04±2.58	12.21±3.75	6.78±5.25	2.33±2.02	2.21±2.35	2.25±1.49
With sons/daughters	20.66±8.49	40.37±7.32	10.41±2.90	10.34±4.40	8.29±2.76	11.37±4.09	10.12±5.13	3.68±1.79	3.51±2.45	2.93±1.49
Alone	24.44±8.93	45.06±8.74	10.44±3.01	13.56±5.14	10.38±1.86	10.50±4.41	9.50±5.06	3.38±1.86	3.19±2.83	2.94±1.29
p	** 0.001**	** 0.001**	0.510	** 0.022**	** 0.001**	0.176	**0.000 **	** 0.001**	** 0.007**	** 0.010**
Illness duration (months)										
<6	29.54±8.81	46.01±7.57	10.69±2.87	13.22±4.20	10.09±2.20	11.99±4.03	5.65±4.32	1.93±1.74	1.81±2.05	1.92±1.29
6-12	21.44±8.23	43.44±7.43	11.34±2.46	11.44±4.30	8.88±2.55	11.75±4.06	9.50±5.11	3.19±1.84	3.28±2.34	3.03±1.36
>12	22.66±9.28	42.88±8.67	10.64±2.79	11.05±4.86	9.52±3.12	11.73±3.77	9.55±5.79	3.48±2.12	3.22±2.75	2.84±1.60
p	**0.000 **	0.057	0.465	**0.010 **	**0.032 **	0.751	**0.000 **	**0.000 **	** 0.001**	**0.000 **
Post-stroke deficits										
None	31.77±7.91	45.77±7.43	10.62±2.77	12.68±3.62	10.03±2.09	12.43±3.76	5.74±4.40	2.13±1.64	1.57±2.06	2.07±1.29
1 deficit	22.23±7.79	44.03±8.21	11.05±2.80	12.05±5.01	9.95±2.73	10.99±3.94	8.60±5.14	3.00±2.15	3.05±2.39	2.55±1.39
More than one	18.11±8.14	41.68±8.72	10.54±2.69	10.57±5.24	7.93±3.15	12.64±3.95	11.04±6.27	3.64±2.20	4.07±2.62	3.29±1.90
p	** 0.000**	0.069	0.475	0.085	** 0.010**	** 0.044**	**0.000 **	**0.003 **	**0.000 **	**0.002 **

p-vap-value based on the Mann-Whitney U test and the one-way ANOVA test. AIS: Acceptance of Illness Scale, PHS: Perception of Health Scale, BHS: Beck Hopelessness Scale

**Table 5:** Linear Regression Analysis of Illness Acceptance Level and Beck Hopelessness Level

Model 1. Illness Acceptance Level Regression Analysis					Model 2. Beck Hopelessness Level Regression Analysis			
Dependent Variable: Illness Acceptance Scale score					Dependent Variable: Beck Hopelessness Scale score			
	β	95% IC	p	R2	β	95% IC	p	R2
MBI	.585	.344 ; .592	**<.001 **		-.115	-.320 ; -.125	** <.001**	
PHS	-.282	-.264 ; -.036	**.010**					
BHS	-.984	-.596 ; -.192	**<.001**					
AIS					-.083	-.329 ; -.121	**<.001**	
(Constant)	1.939	7.332 ; 23.696	**<.001**	.509	.994	19.661 ; 26.037	**<.001**	.410

95% IC: 95% Confidence Interval for β. MBI: Modified Barthel Index, PHS: Perception of Health Scale, BHS: Beck Hopelessness Scale. AIS: Acceptance of Illness Scale

## Discussion

This study found that the total scores of the scales vary significantly based on participants' demographic characteristics. A significant relationship was also observed between participants' illness acceptance, health perception, and hopelessness levels. Particularly, the sudden onset of a chronic illness is a challenging process, making coping difficult^8^,^36^. Illness acceptance positively affects the development of health behaviors, an important factor for minimizing complications^16^. In this study, participants' illness acceptance is moderate. A limited number of studies on illness acceptance in stroke survivors were found in the literature^25^. Consistent with our findings, Guzek and Kowalska also reported moderate illness acceptance in stroke survivors^25^. In order to improve care quality and provide holistic care, it is essential to consider illness acceptance as it significantly affects the diagnosis, treatment, and rehabilitation processes of stroke survivors. 

Acquisition, maintenance, and improvement of health behaviors in chronic illness management are directly related to health perception. Individuals with a positive health perception are more willing to take responsibility for their health and healthcare services^17^,^18^. In this study, the participants' mean total health perception score was 50.30±0.59. Among the sub-dimensions, the center of control and the importance of health had the highest scores. This may be explained by stroke survivors’ desire to maintain control over their health, recognition of the importance of healthy living behaviors, and willingness to regain their former physical strength, as most participants were moderately dependent and wanted to avoid long-term dependence. In the literature, there are no studies examining health perception in stroke survivors. However, Gür and Sunal reported a mean health perception of 47.37±5.77 in individuals with coronary artery disease^37^. Since the scale has no cut-off points, direct comparisons of health perception levels cannot be made. 

Hope is another important factor influencing an individual's health. It increases individuals' compliance with illness treatment, whereas worsening physical condition, pain, and other post- stroke complications may lead to hopelessness^18^,^20^. In this study, participants showed a mild level of hopelessness. The literature contains limited research on hopelessness levels among stroke survivors^20^. However, Aisy and Darliana found that stroke survivors showed moderate levels of hopelessness^20^. This difference may be attributed to differences in participants’ levels of dependence between the two studies. 

In this study, the relationship between illness acceptance and demographic variables was examined, revealing a negative correlation between participants' age and illness acceptance. This may be attributed to older individuals’ reluctance to become dependent on others. Previous studies support this finding^12^,^36^. A positive correlation was found between participants' level of education and illness acceptance. Guzek and Kowalska reported similar findings among stroke survivors, while Baczewska et al. and Şireci and Karabulutlu also reported that individuals' illness acceptance increased with higher education levels in different chronic illnesses^12^,^25^,^38^. This may be explained by the fact that higher education levels increase individuals' awareness, information- seeking behavior, and access to information. Similar to this study, Białek and Sadowski found that married individuals exhibited higher illness acceptance^36^. The high rate of illness acceptance among married individuals may be due to the support provided by a spouse, which can facilitate coping with the illness, overcoming problems, and adapting to limitations. 

This study found that employed individuals exhibited higher illness acceptance. Similar findings have been reported in other studies^36^,^38^,^39^. Joblessness or job loss due to illness-related limitations negatively impacts illness adaptation^38^. Similarly, Białek and Sadowski found that individuals living with their families were more willing to accept their illness^36^. This supports the idea that there is a positive relationship between the presence of a support system and adaptation to limitations in the illness process, coping with problems, and illness acceptance. Another result of this study is that acceptance of the illness decreases as more time elapses after diagnosis. Studies conducted with different chronic diseases support this result^10^,^12^,^36^, suggesting that prolonged illness duration increases an individual's exposure to and struggles with illness-related negativism, ultimately leading to lower illness acceptance. 

A negative relationship was found between post-stroke deficits and illness acceptance. Studies show that post-illness deficits negatively impact illness acceptance^10^,^39^. Additionally, as the participants' independence levels increased, their levels of illness acceptance also improved. Furmańska et al. found that illness acceptance decreased as disability increased in individuals with multiple sclerosis^40^. These findings suggest that there is a positive relationship between stroke survivors’ ability to perform ADLs independently and their level of illness acceptance. 

Another variable examined in the study was health perception. As the participants' age increases, their health perceptions decrease. This may be related to younger people's desire to seek information and take responsibility for their health. In the study, a positive relationship was found between education level and health perception. This may be attributed to the fact that individuals with higher education levels tend to have a stronger drive for information-seeking and awareness. Unlike our study's findings, Gür and Sunal found no relationship between these variables and health perception^37^. 

This study also examined the relationship between participants' hopelessness levels and demographic variables. A positive correlation was found between participants' age and hopelessness levels. Studies conducted with different chronic illnesses support this finding^41^,^42^, suggesting a negative relationship between aging and hope levels. Additionally, illiterate participants exhibited higher levels of hopelessness. Similarly, previous studies have shown that hopelessness decreases as education level increases^41^–^43^. This may be attributed to the fact that individuals with higher education levels are more likely to research their illnesses, adopt a more questioning attitude, and maintain a greater sense of hope. In contrast to these results, Erdoğan et al. reported that individuals with postgraduate education exhibited the highest levels of hopelessness^42^. 

This study found that single individuals exhibited higher hopelessness levels. Kılınç et al. and Erdoğan et al. reached a similar result that supports this finding^42^,^44^. This result may be attributed to stronger support systems for married individuals, which can help mitigate feelings of hopelessness. Additionally, studies indicate that unemployed participants experience higher hopelessness levels^41^,^43^. This suggests that joblessness due to chronic illness negatively impacts individuals’ sense of hope. This study also revealed that individuals living alone had higher hopelessness levels. Similarly, Atan et al. found that cancer patients living alone exhibited higher levels of hopelessness^45^. This may be explained by the reduced social support of individuals living alone, which can have a negative impact on hope. 

Furthermore, a positive correlation was observed between illness duration and hopelessness. Studies conducted with different chronic illnesses also support this finding^42^,^43^. This may be attributed to the fact that prolonged illness duration led to longer individuals’ exposure to illness- related negativism. Another key result is that as participants' ability to act independently increases, their hopelessness levels decrease. Similarly, Aisy and Darliana reported that hopelessness levels increased as the dependence levels increased^22^. These results suggest a positive relationship between hope and an individual’s ability to perform ADLs independently. 

Illness acceptance, health perception, and hope levels play an important role in the treatment and rehabilitation process of stroke survivors, yet they are not widely discussed. These key points should be considered by nurses providing care in clinical settings. Incorporating these parameters into nursing care could improve individuals' adherence to the illness treatment and health personnel recommendations while also contributing to developing and maintaining healthy living behaviors^17^,^18^,^22^. The study findings indicate a significant relationship between illness acceptance, health perception, and hopelessness. However, when the literature was reviewed, a limited number of studies examining these relationships in stroke survivors were found. Therefore, the data obtained in this study are expected to bridge this gap in the literature and provide guidance for nurses in caring for stroke survivors. 

**Limitations and strengths of the study**

The study results are limited by a small sample size, single-time measurement, and data collection from a single study center. Therefore, it is not possible to generalize the results to all stroke survivors. In addition, individuals' dependence levels were not equally collected, which may also be considered a limitation. Another limitation is that the effect of the studied parameters on nursing care was not examined. Furthermore, since this study is cross-sectional, it does not allow for causal inferences. Despite these limitations, this study is one of the pioneering studies in the literature to draw attention to the levels of acceptance, positive health perception, and hope in stroke survivors. Future studies should explore the effects of these parameters on nursing intervention outcomes. In this regard, this study serves as a guide for future research in this area. 

## Conclusions

This study determined that illness acceptance, health perception, and hopelessness levels in stroke survivors were influenced by sociodemographic variables and illness characteristics. Additionally, a significant relationship was observed between illness acceptance, health perception, and hopelessness levels. Nursing care should be planned accordingly to provide holistic and quality care to stroke survivors during the treatment and care process. During the processes of illness acceptance, positive health perception, and development of hope, nurses should engage in open communication with patients, allowing them to express their doubts, fears, and expectations. They should also assist individuals in developing effective coping mechanisms; they should meet their information needs and design suitable nursing interventions. Therefore, further research is recommended to explore the levels of illness acceptance, health perception, and hopelessness in stroke survivors to inform and improve nursing interventions. 
